# Decision tree-Markov model of perinatal depression screening: a cost–utility analysis

**DOI:** 10.3389/fpubh.2024.1308867

**Published:** 2024-05-20

**Authors:** Yehuan Yang, Ruimin Zheng, Li Yang, Xing Huang, Tong Zhang

**Affiliations:** ^1^National Center for Women and Children's Health Chinese Center for Disease Control and Prevention, Beijing, China; ^2^Capital Institute of Pediatrics, Beijing, China

**Keywords:** perinatal depression, perinatal psychiatric disorders, depression screening, decision tree-Markov model, cost-utility analysis

## Abstract

**Background:**

Perinatal depression affects the physical and mental health of pregnant women. It also has a negative effect on children, families, and society, and the incidence is high. We constructed a cost–utility analysis model for perinatal depression screening in China and evaluated the model from the perspective of health economics.

**Methods:**

We constructed a Markov model that was consistent with the screening strategy for perinatal depression in China, and two screening strategies (screening and non-screening) were constructed. Each strategy was set as a cycle of 3 months, corresponding to the first trimester, second trimester, third trimester, and postpartum. The state outcome parameters required for the model were obtained based on data from the National Prospective Cohort Study on the Mental Health of Chinese Pregnant Women from August 2015 to October 2016. The cost parameters were obtained from a field investigation on costs and screening effects conducted in maternal and child health care institutions in 2020. The cost–utility ratio and incremental cost–utility ratio of different screening strategies were obtained by multiplicative analysis to evaluate the health economic value of the two screening strategies. Finally, deterministic and probabilistic sensitivity analyses were conducted on the uncertain parameters in the model to explore the sensitivity factors that affected the selection of screening strategies.

**Results:**

The cost–utility analysis showed that the *per capita* cost of the screening strategy was 129.54 yuan, 0.85 quality-adjusted life years (QALYs) could be obtained, and the average cost per QALY gained was 152.17 yuan. In the non-screening (routine health care) group, the average cost was 171.80 CNY per person, 0.84 QALYs could be obtained, and the average cost per QALY gained was 205.05 CNY. Using one gross domestic product *per capita* in 2021 as the willingness to pay threshold, the incremental cost–utility ratio of screening versus no screening (routine health care) was about −3,126.77 yuan, which was lower than one gross domestic product *per capita*. Therefore, the screening strategy was more cost-effective than no screening (routine health care). Sensitivity analysis was performed by adjusting the parameters in the model, and the results were stable and consistent, which did not affect the choice of the optimal strategy.

**Conclusion:**

Compared with no screening (routine health care), the recommended perinatal depression screening strategy in China is cost-effective. In the future, it is necessary to continue to standardize screening and explore different screening modalities and tools suitable for specific regions.

## 1 Introduction

Perinatal depression/perinatal psychiatric disorders (PND) include postpartum depression (PPD). PPD occurs from the beginning of pregnancy up to 12 months after delivery (1), and the core symptoms are low mood, loss of interest or pleasure, and decreased energy, often accompanied by decreased concentration, low self-evaluation, and suicidal tendencies. Internationally, the total prevalence rate of depression among pregnant and postpartum women is 11.9 to 17.0% (2, 3) and is on the rise (4). A meta-analysis in China showed that the total prevalence of perinatal depression was 16.3%, prenatal depression accounted for 19.7%, and PPD accounted for 14.8%, and it continued to rise in the past decade (5). With the implementation of the comprehensive “two-child” and “three-child” policies in China, a large number of pregnant women over 35 years of age will be added in China, and this age group is at a higher risk of depression (6).

If pregnant women at risk of depression do not get timely and effective help, the depression during this period can have a huge ripple effect (7). During maternity, it can interfere with the mother’s daily life, leading to worsening symptoms and prolonged depression. When depression occurs or extends into the period after childbirth, it can also affect a mother’s ability to care for her baby and other children in the family, as well as a woman’s relationship with her partner. The most serious short-term outcome of depression during pregnancy is self-injury or suicide (8). Studies have shown that 28% of maternal deaths are because of suicide (9), which is the second most common cause of death related to pregnancy and childbirth.

The disease burden related to PND to pregnant women, their children, families, and society is huge and constantly rising (10), and it cannot be ignored. A UK report (11) has shown that depressed women spend 87% more on medical services than non-depressed women. In the UK, the total lifetime cost per depressed mother is estimated to be £75,728. If prevalence estimates are applied, the combined cost of maternal anxiety and depression is approximately £8,500 per woman who gives birth, resulting in a total cost of £6.6 billion in the UK (12). Tracking the knock-on effects in public sector costs, the cost per child of a mother with PND exceeds £3,030 (2010–2011 prices), the cost of reduced household income is £1,400, and the cost of health-related quality of life loss is estimated at £3,760. These reports highlight the widespread and lasting consequences of PND for women, their families, and society (11).

Screening itself is a kind of health promotion activity, and carrying out screening for depression during pregnancy has important public health relevance and great influence. Existing studies have shown that by adding simple and highly specific screening items and interventions for pregnant women to routine maternal health care, early detection and intervention for PND can significantly reduce the incidence of perinatal depression, improve the referral rate and service utilization rate (13), obtain positive mental health results, improve the outcome of parenting, and reduce the occurrence of major depressive disorder. From a societal perspective, high-risk mothers with major depression incur greater health care and workplace productivity costs than non-depressed mothers. Identifying and effectively treating mothers at high risk of depression can save substantial costs for public insurers and employers (14). Therefore, it is necessary to explore whether psychological screening including depression should be conducted regularly during pregnancy and the perinatal period. Providing screening services for pregnant women with perinatal depression may have societal benefits because pregnant women with perinatal depression have higher medical costs than those without perinatal depression.

Health economic evaluation of perinatal depression screening is mainly based on cost–benefit analysis and cost-effectiveness analysis (15). The results show that simple scale screening may not be cost-effective (16); however, screening and intervention/treatment can effectively reduce depression compared with no screening (routine health care) and is cost-effective in the short term (16–21). No previous studies in China have investigated the cost-effectiveness of screening for perinatal depression; therefore, the benefits are unknown.

The China maternal mental health management expert consensus (22) considered international experience and the policy environment and situation in China. It recommended use of the self-assessment Edinburgh Postpartum Depression Scale (EPDS)/PHQ-9 in the first trimester, second trimester, third trimester, and 42 days after delivery, as a screening strategy to assess maternal mental health four times. This study focused on research evaluation from the perspective of payment and the health care system, so that the findings are relevant to clinical decision-makers. The costs did not include direct non-medical costs, indirect costs, and hidden costs.

## 2 Methods

### 2.1 Model overview

We used TreeAge Pro software to build decision tree-Markov models, monitor transitions between discrete health states, and specify the impact of interventions on transition probabilities. We constructed a decision tree-Markov model for two screening strategies. We assumed that pregnant women entered the study cohort during early pregnancy and were divided into two groups: the screened group and the unscreened group (routine care). The simulation ended when 3 months postpartum or death was observed. The health status of pregnant women during the depression screening process was divided into four states: normal, suspicious, high risk, and death. The screening group referred to perinatal depression screening, which occurred at four time points (in the first trimester, the second trimester, the third trimester, and 42 days after delivery). In the simulation, if women were classified as having perinatal depression status using the depression scale score, they were transferred for timely intervention and treatment. In the group without screening (routine care), routine maternity care was carried out. High-risk groups with more serious symptoms received intervention treatment at specialized hospitals.

### 2.2 Resources and costs

In the National Prospective Cohort Study on the Mental Health of Chinese Pregnant Women (NSMCP), the natural conditions of perinatal depression during pregnancy and the perinatal period in China were described, and the positive detection rate of perinatal depression in each period was calculated according to the scale scores of pregnant women in different time periods. The change in depression outcome rates in the second and third trimesters and the postpartum period were compared with the depression level in the baseline period (early pregnancy). NSMCP was jointly established by the Maternal and Child Health Center of the Chinese Center for Disease Control and Prevention and five maternal and child health institutions: Beijing Haidian District Maternal and Child Health Care Hospital, Shanxi Provincial Maternal and Child Health Care Hospital, Jilin Provincial Maternal and Child Health Care Hospital, Zhuhai Maternal and Child Health Care Hospital of Guangdong Province, and Shenzhen Maternal and Child Health Care Hospital of Guangdong Province. From August 1, 2015 to October 31, 2016, pregnant women were recruited and their psychological status was followed up at seven time points during pregnancy and the perinatal period. A self-filled questionnaire and the EPDS survey were used to obtain the general demographic characteristics and depression status of the pregnant women. In this part, EPDS scores at 13 weeks of gestation were used to represent depression in the first trimester, the average EPDS scores at 17 weeks and 24 weeks of gestation were used to represent depression in the second trimester, and the average EPDS scores at 31 weeks and 37 weeks of gestation were used to represent depression in the third trimester. The mean scores of EPDS at 3 and 42 days postpartum were representative of PPD.

Screening cost research sites were selected from typical areas in eastern, central, and western China that have carried out maternal and perinatal depression screening ≥2 times and have clear screening fee standards. The sites included Beijing Haidian District Maternal and Child Health Care Hospital, Beijing Fengtai District Maternal and Child Health Care Hospital, Shanxi Province Maternal and Child Health Care Hospital, and Chongqing Maternal and Child Health Care Hospital. From November 1, 2019 to October 30, 2020, women registered with Beijing Haidian Maternal and Child Health Hospital who had completed all four time points of psychological scale screening were selected as research subjects, and the screening results were retrieved with screening software. Outcome rate analysis was then conducted. The direct economic costs of pregnancy depression screening at institutions were collected through interviews with the principal person in charge of the department of cardiology or other doctors using the screening cost questionnaire.

PubMed, Bailian, Wanfang database, and China National Knowledge Infrastructure were searched for the following terms: “perinatal AND depression AND health economics” and “perinatal depression OR postpartum depression (postpartum depression) AND cost-effectiveness analysis OR cost-utility analysis.” These terms were used to search for health economics studies related to depression during pregnancy without any time limitation.

### 2.3 Screening and intervention costs

We used a willingness to pay (WTP) threshold related to gross domestic product (GDP) *per capita* to select the most cost-effective strategies, as recommended by the World Health Organization. The *per capita* GDP of China in 2021 (RMB 80,976) published in the Statistical Bulletin of National Economic and Social Development of the People’s Republic of China (23) was taken as the value of per capita GDP used in this study.

As the current screening strategy is integrated into routine maternity and perinatal health care, no additional indirect costs such as transportation, accommodation, or missed work were generated because of the screening of depression during pregnancy. The positive rate of daily screening in health institutions is low, and indirect and invisible costs are not easily obtained; therefore, this study did not include them and only calculated the direct medical burden based on outpatient information. The direct screening cost of the screening group is all the costs directly used for the screened population, including the costs required by the screening project and the costs required by the treatment after the detection of the affected population, including screening costs, registration costs, consulting costs, treatment costs, and medical costs. However, the cost of the unscreened group (routine care) only includes the cost of the treatment of the active healers. We assumed that only the patients with severe depression or perceived symptoms would actively seek medical treatment, so used the direct medical expenses of psychiatric hospitals in the literature as a substitute. Given that perinatal depression mortality is rarely reported, this study used the 4% suicide rate for depression in the general population published on the World Health Organization website instead of mortality.

### 2.4 Screening sensitivity and specificity

Not all high-risk patients received treatment, and the positive predictive value of the scale in the literature was uniformly used in the calculation [EPDS: 59.00%; PHQ-9: 58.40% (24)], that is, the proportion of the real number of patients who were screened positive by the scale.

### 2.5 Utilities and quality-adjusted life years

In this study, quality-adjusted life years (QALYs) were used as health utility indicators, ranging from 0 to 1, where 0 represents no health and 1 represents complete health. In view of the lack of published research on QALYs in depressed pregnant women in China, the QALYs of pregnant women in different depressive states refer to international reports (25–27) and set the QALYs of normal pregnant women as 0.86 (0.77–0.95). The QALYs of suspicion for a depressive state (the lower threshold of depression diagnosis) were set at 0.80 (0.72–0.83), the high-risk state was set at 0.63, and the range of QALYs fluctuated greatly according to the degree of depression (0.30–0.73). Death was set at 0.

### 2.6 Prevalence and transition probabilities

In this study, the initial probability and transition probability of the unscreened natural population (routine care) were obtained by analyzing NSMCP data. Maternal data obtained from the field survey involving the screening strategy for perinatal depression were analyzed and processed to obtain the initial probability and transition probability of the screening group.

### 2.7 Outcomes

Primary outcomes of screening strategies were evaluated using the cost–utility ratio (CUR) and the incremental CUR (ICUR), and methods and outcomes were reported in accordance with the Comprehensive Health Economic Evaluation Reporting Standards. The lower the CUR and ICUR values, the higher the health economic effectiveness of the screening strategy. CUR is a measure that compares cost and QALYs, and indicates the cost per QALY. ICUR represents the increased cost of each additional unit of QALY, reflecting the unit cost of the utility difference between the two strategies.

### 2.8 Sensitivity analysis

To reflect the uncertainty around ICURs, sensitivity analyses were conducted to simulate unidirectional certainty and probability (one-way deterministic and simulated probabilistic sensitivity analyses). Difference discount was used in sensitivity analysis, which was calculated according to the discount rate of 3% recommended by the World Health Organization, and the sensitivity analysis range was 0–7%. All costs were calculated in RMB (unit: yuan), and the research time was short and the survey time was recent, so the discount issue was not considered. However, the direct medical cost data of specialized hospitals came from literature reports in 2011, so the discount rate was used to convert the data. The upper and lower limits used in the one-way probability sensitivity analysis were used to define the distribution parameters, and the mean and range of fluctuations were calculated from the data obtained from the survey. The prevalence of perinatal depression/screening positive rate was used as the upper and lower limits of one-way deterministic sensitivity analyses with 95% confidence intervals. The factors to which ICURs are sensitive were ranked from highest to lowest using tornado plots.

The transition probability, health utility value, cost, and other parameters of the Markov model were set according to their respective distribution (Table 1). Monte Carlo simulation was performed on n = 1,000 samples to conduct probabilistic sensitivity analysis of the Markov model for different screening strategies. Considering the limitation of parameter sources and data volume, the parameter distribution was adopted as the default distribution for health economics research, that is, the QALY value was set as Beta distribution and the cost was set as Gamma distribution.

**Table 1:** Monte Carlo simulation parameters (*n* = 1,000).

Indicators	Mean	Standard deviation	Reference probability distribution
QALY (high risk)	0.63	0.15	Beta
QALY (suspicious)	0.80	0.08	Beta
QALY (normal)	0.86	0.09	Beta
Screening cost (yuan/time)	19.00	21.83	Gamma
First diagnosis fee (yuan/time)	42.50	10.85	Gamma
Psychiatric registration (yuan/time)	22.50	8.66	Gamma
Treatment frequency (times)	3.33	0.84	Gamma
Treatment cost (yuan/time)	229.00	209.41	Gamma

QALY, quality-adjusted life years.

### 2.9 Ethics approval

This study was approved by the Ethics Committee of the Maternal and Child Health Center, Chinese Center for Disease Control and Prevention on March 29, 2021 (batch number: FY2021-03).

## 3 Results

### 3.1 Perinatal depression status in the NSMCP cohort

**Figure 1:**
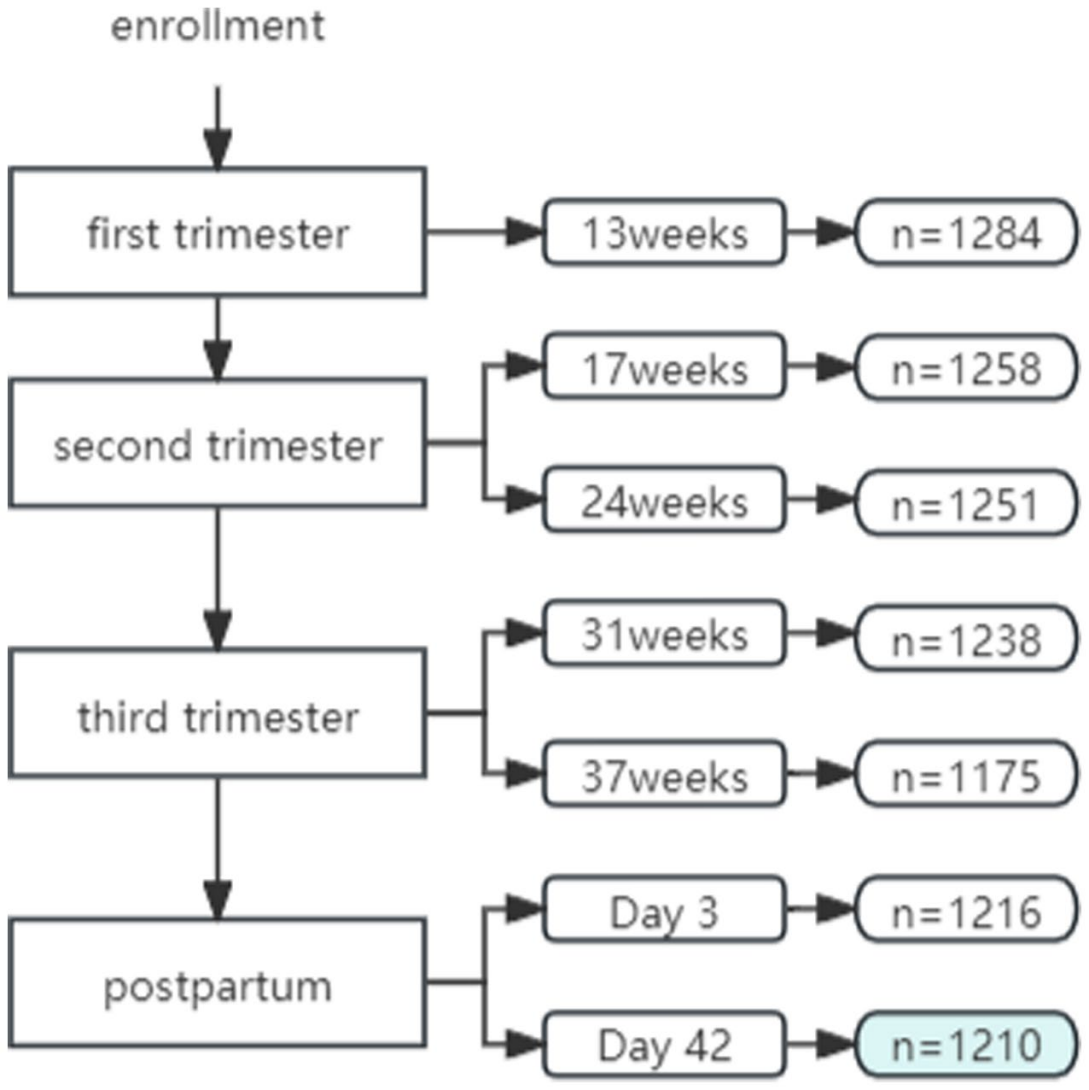
Cohort study of enrollment.

A total of 1,284 women were recruited from the NSMCP cohort. A total of 1,210 women were included in the analysis (240 in Beijing, 250 in Shanxi, 223 in Jilin, 251 in Zhuhai, and 246 in Shenzhen) who had completed at least six follow-up visits by postnatal day 42 (D42). The follow-up rate was 94.2% (1,210/1,284) (Figure 1).

The average scores of EPDS were 6.43 ± 3.42, 6.01 ± 2.85, 5.80 ± 2.92, and 5.93 ± 3.15 in the first trimester, the second trimester, the third trimester, and postpartum, respectively. The score of EPDS was the highest in the first trimester, decreased in the second and third trimester, and increased postpartum. The detection rates of depressive symptoms (≥9 points) in each period were 26.5, 18.0, 15.6, and 18.1%, respectively, and the differences were statistically significant (*p* < 0.05).

The depression status of each group in the baseline period (early pregnancy) was used as the benchmark, and the changes of depression status in middle and late pregnancy and postpartum were compared and are summarized in Table 2. According to the scores of the scale in different periods, the detection rate of depressive symptoms of pregnant women in each period was calculated. The results showed that 62.8% of pregnant women had normal scores in the first, second, and third trimesters of pregnancy and postpartum, that is, 37.2% of the pregnant women had at least one positive result of depression during pregnancy, and 6.94% of them had persistent positive results of depression.

**Table 2:** Changes in baseline subjects in each group according to EPDS scores in three periods during pregnancy and the postpartum period.

Baseline period (first trimester)	Classification	Second trimester	Third trimester	Postpartum
Normal group (*n* = 884)	0–9	826	848	823
	9–13	19	30	46
	≥13	3	3	11
Suspicious group (*n* = 236)	0–9	158	176	173
	9–13	74	53	52
	≥13	4	7	11
High-risk group (*n* = 93)	0–9	48	53	52
	9–13	31	27	29
	≥13	14	13	12

EPDS, edinburgh postpartum depression scale.

In the normal group, only 3.4–4.2% of pregnant women would have had suspicious positive results during follow-up and 0.3–1.2% of the pregnant women would have directly progressed to high-risk status at a certain period. Most of the pregnant women (93.1–96.0%) would have maintained normal levels. In the suspicious positive group, 67.0–74.6% of the pregnant women recovered from depression during the follow-up period. However, 1.7–4.7% of the pregnant women had worsening symptoms during the follow-up period of pregnancy, including 1.7% in the second trimester, 3.0% in the third trimester, and 4.7% postpartum. In the high-risk group, 29.0–33.3% of the pregnant women’s symptoms decreased to suspicious severity during the follow-up, and for 51.6–57.0% of the pregnant women, depressive symptoms disappeared. The percentage of pregnant women with positive detection of depression (≥9 points) in the second and third trimesters and postpartum was 16.7% (55/329), and 30.7% (101/329) of pregnant women had positive detection of PPD.

The natural outcome analysis between the baseline period and the last observation point (42 days postpartum) showed that 9.4% of the normal group had suspected depressive symptoms. In the suspicious positive group, 86.0% spontaneously improved, and only 14% still had suspected depression. In the high-risk group, 37.9% of the patients’ symptoms were relieved to suspicious, and 62.1% of the patients were completely improved and symptoms disappeared. Most of the women were detected as positive during pregnancy, and only 4.88% were detected as positive for the first time postpartum. The specific natural transition probabilities of perinatal depression at different time points during pregnancy and childbirth are shown in Table 3.

**Table 3:** Transition probability (%) of a depression state in each early pregnancy.

Outcome	First trimester	Upper limit	Lower limit	Second trimester	Third trimester	Postpartum
Normal → normal	72.80	75.30	70.30	93.40	95.90	93.10
Normal → suspicious				2.10	3.40	5.20
Normal → high risk				0.30	0.30	1.20
Suspicious → normal	19.50	21.70	17.30	66.90	74.60	73.30
Suspicious → suspicious				31.40	22.50	22.00
Suspicious → high risk				1.70	3.00	4.70
High risk→ normal	7.70	9.20	6.20	51.60	57.00	55.90
High risk→ suspicious				33.30	29.00	31.20
High risk→ high risk				15.10	14.00	12.90

### 3.2 Perinatal depression status in the screening group

The survey found that a total of 17,005 pregnant women underwent perinatal depression screening in Beijing Haidian Maternal and Child Health Hospital from November 1, 2019 to October 30, 2020. Among them, 589 pregnant women who accepted the perinatal depression screening strategy and completed all four perinatal depression screenings were included in the analysis. The positive rate of screening was 28.5% in the first trimester, 4.7% in the second trimester, 3.9% in the third trimester, and 3.4% postpartum, and the specific outcomes are shown in Table 4.

**Table 4:** State transition probability (%) of the screening group.

Outcome	First trimester	Upper limit	Lower limit	Second trimester	Third trimester	Postpartum
Normal → normal	71.50	72.50	67.80	97.10	96.40	96.90
Normal → suspicious				2.60	3.10	1.40
Normal → high risk				0.20	0.50	1.70
Suspicious → normal	26.50	27.50	22.90	91.00	94.90	95.50
Suspicious → suspicious				7.10	5.10	2.60
Suspicious → high risk				1.90	0	1.90
High risk → normal	2.00	3.20	0.90	83.30	100.00	100.00
High risk → suspicious				8.30	0	0
High risk → high risk				8.30	0	0

### 3.3 Cost analysis of perinatal depression screening

In this study, it was assumed that real patients were treated with the same regimen. At the same time, the study assumed that all pregnant women in the unscreened group would only seek medical treatment when they had serious symptoms, so the treatment costs of pregnant women in the unscreened group were replaced by the direct medical expenses of specialized hospitals in the literature (28–30). In the investigation of institutions, it was found that most of the suspected positive screening cases were referred to the psychological department for a simple interview, which generally did not incur treatment costs. Certain antidepressant drugs are rarely used during pregnancy because of safety issues related to breastfeeding after taking them, as verified by a literature review (31) and interviews with psychiatrists in maternal and child health care hospitals. The proportion of PPD medication is about 50–60%, and the commonly used drugs are sertraline, paroxetine, and escitalopram. Therefore, in this study, the price of sertraline was taken as the representative, and the medication proportion was set as 50%. The drug cost of this proportion of postpartum women who took medication was calculated, and the fluctuation range was the cost of two other drugs that might be used.

Normal screening cost = screening cost = 19 yuan; suspicious cost = screening fee + registration fee + first visit fee = 84 yuan; screening positive cost = screening cost + registration cost + first visit cost + treatment cost × number of treatments × positive predictive value of the scale + drug cost × 0.5 = 820.32 yuan. Positive cost of no screening (routine care) = direct medical cost of specialized hospitals (discounted) × positive predictive value of the scale = 829.48 yuan. See Table 5 for specific cost data and sources.

**Table 5:** Cost parameters (yuan).

Non-unit level cost	Mean value	Lower limit	Upper limit	Data sources
Screening cost	19.0	5.0	51.0	Field investigation
Registration fee	22.5	15.0	30.0	
First visit fee	42.5	30.0	49.5	
Treatment cost	229.0	79.0	495.0	
Treatment times	3	2	12	
Drug cost (3 months)	479.7	97.2	1,106.1	Drug guidelines, field investigation
Cost of specialist hospital treatment	1,405.9	846.8	1,702.0	Literature

### 3.4 Cost-effectiveness analysis of screening strategies for perinatal depression

The decision tree-Markov model for screening strategies for pregnancy and perinatal depression constructed in this study was analyzed by backfold. Then, under the condition of set WTP threshold standard (1 × GDP = 80,976), the two strategies of screening and non-screening (routine care) were analyzed by cost-effectiveness, and then the two strategies were compared by ICUR (Table 6).

**Table 6:** Cost–utility analysis of different screening strategies (*per capita*).

Screening strategy	Cost (RMB)	Incremental cost (RMB)	Utility (QALYs)	Incremental utility (QALYs)	ICUR	Avg CU
Screening	129.54	NA	0.85	NA	NA-	152.17
Routine care	171.80	42.25	0.84	−0.01	−3,126.77	205.05

Utility is the per capita quality-adjusted life years (QALY) of each group, cost is the per capita cost of each group, ICUR is the incremental cost utility ratio, and Avg CU is the average cost per QALY obtained by each group.NA (not applicable).

Cost–utility analysis showed that the *per capita* cost of the screening group was 129.54 yuan, 0.85 QALYs could be obtained, and the average cost of each QALY was 152.17 yuan. The average cost per person for the unscreened (routine care) group was 171.80 yuan, 0.84 QALYs were available, and the average cost per QALY was 205.05 yuan.

**Figure 2:**
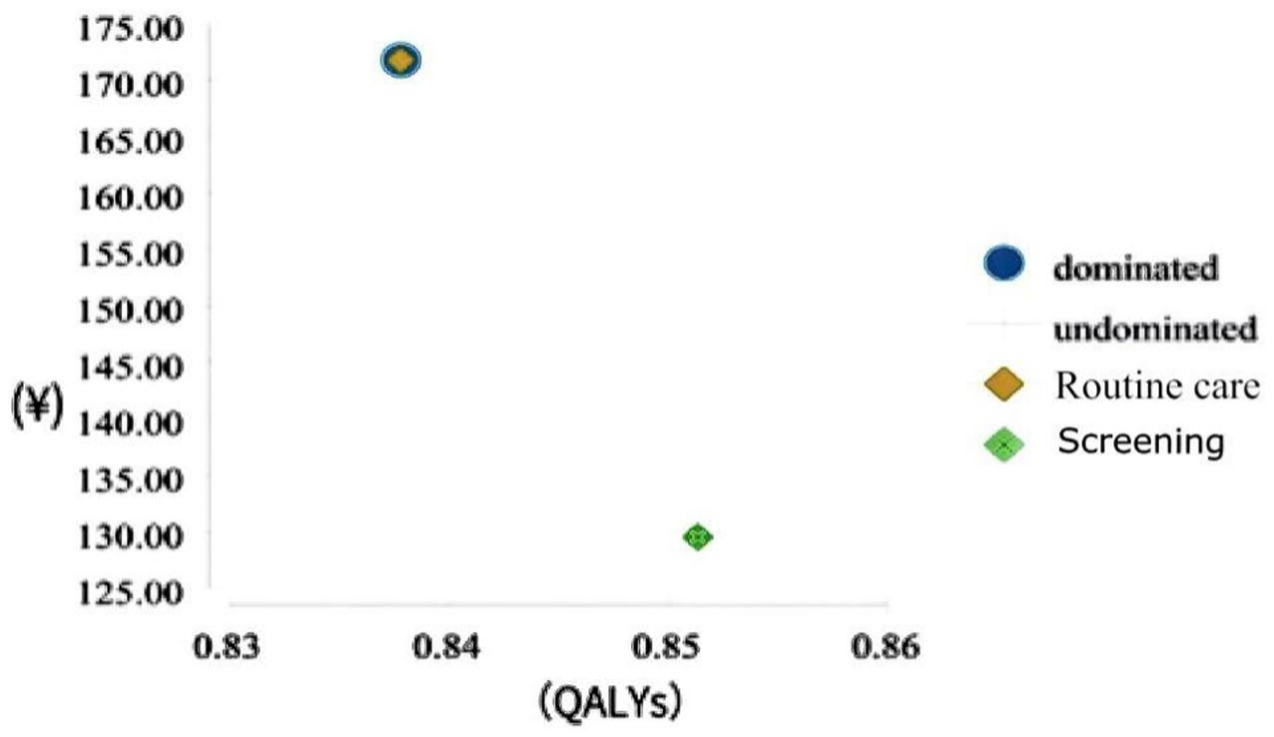
Cost-utility coordinates for different strategies. QALYs, quality-adjusted life years.

Compared with one time of China’s *per capita* GDP (80,976 yuan) in 2021, the ICUR of screening is about −3,126.77 yuan compared with that of non-screening (conventional health care), which is less than one time of *per capita* GDP. Therefore, compared with standard treatment, screening has high cost-effectiveness and is an absolute advantage strategy (Figure 2).

## 4 Sensitivity analysis

### 4.1 Single factor sensitivity analysis

**Figure 3:**
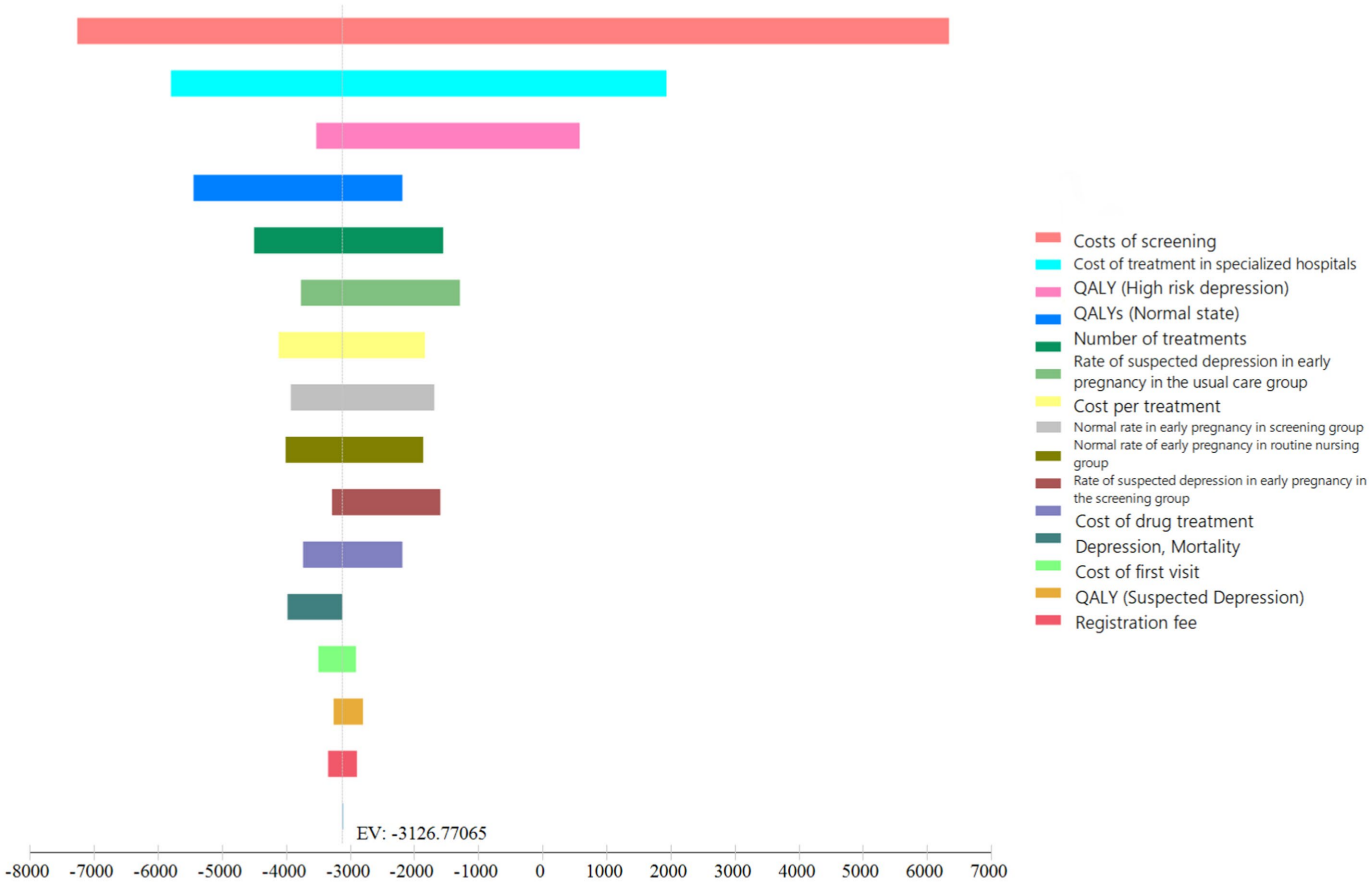
Results of the sensitivity analyses of the decision tree. QALY, quality-adjusted life years.

According to the baseline value and sensitivity analysis range set for the parameters required by the model described above, the sensitivity analysis of the parameters (transfer probability, health utility value, and cost) entered into the Markov model was carried out. The hurricane chart is displayed in Figure 3. The abscissa in the chart represents the expected value or incremental value. The specific bar chart represents the result of the expected value changing with the change of each model parameter value.

The influencing factors of screening strategies in order of their impact (starting with the highest impact) were as follows: screening cost, specialized hospital treatment cost, QALY (high-risk state), QALY (normal state), treatment frequency, early suspected positive rate in the non-screened (routine health care) group, treatment cost (single visit), early normal rate in the screening group, early normal rate in the non-screened (routine health care) group, early suspected positive rate in the screening group, drug cost, depression mortality rate, first visit cost QALY (suspicious status), and registration fee. The screening fee and specialized hospital treatment fee have a significant impact on the results, but the range of changes in all factors does not exceed one time the *per capita* GDP (80,976 yuan) and does not change the choice of the optimal strategy (Figure 3).

### 4.2 Monte Carlo probability sensitivity analysis results

The Monte Carlo simulation results show that the average cost of the screening group is 112.95 yuan and the average utility obtained is 0.85 QALYs. The average cost of the non-screened group was 171.80 yuan and the average utility obtained was 0.83 QALYs. The utility estimated by the Monte Carlo simulation method is close to the point estimation obtained by the queue simulation method, while the average cost of the screening group is reduced and the incremental cost is increased.

**Figure 4:**
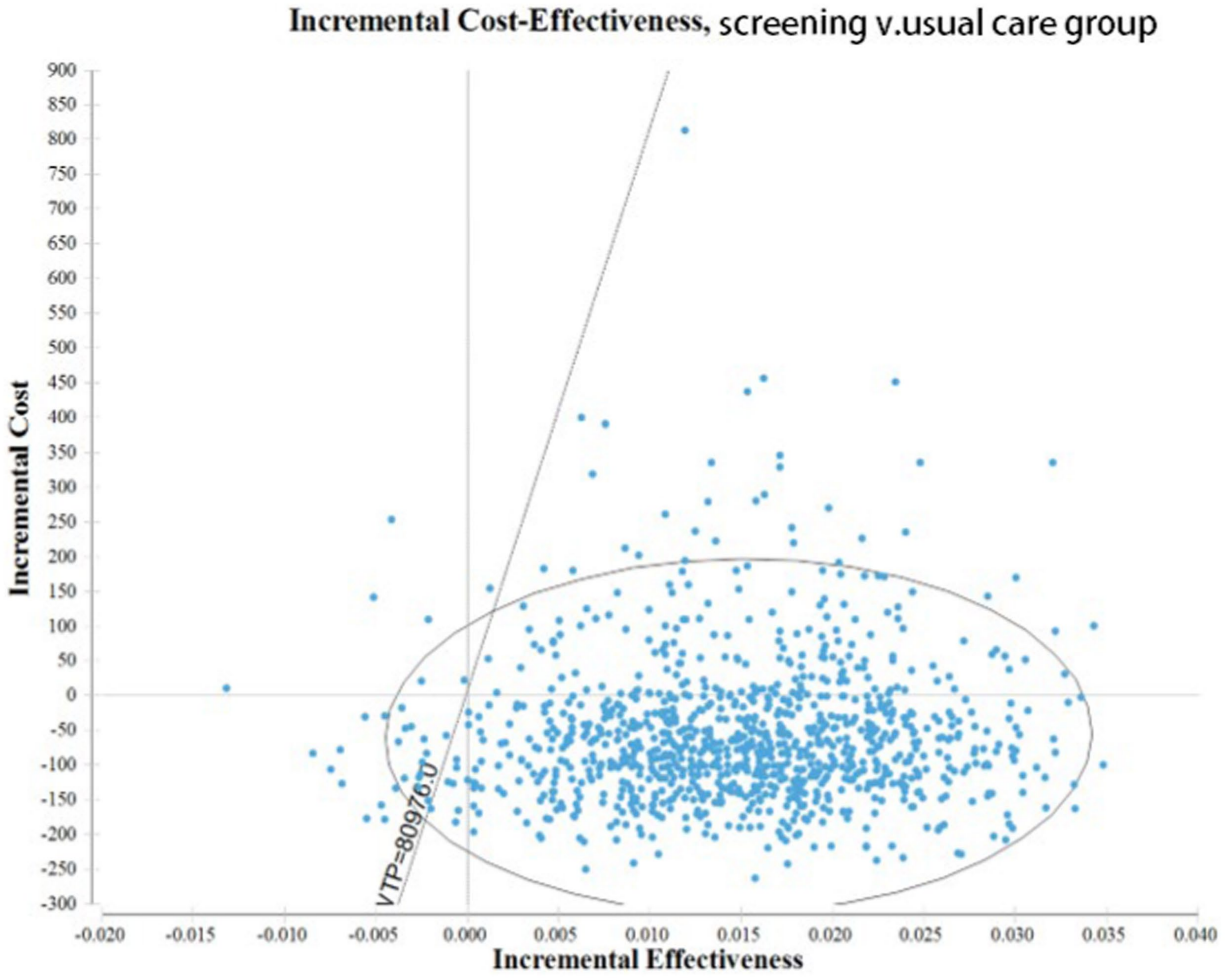
Cost-effectiveness plane showing the scatterplot of the probabilistic.

A multi factor sensitivity analysis was conducted on the constructed strategy model and a 95% confidence interval ICUR scatter plot was obtained (Figure 4). The ICUR scatter chart is distributed in all four phenomena, but most of them are distributed in the fourth quadrant, namely the absolute advantage quadrant, which means that screening is low cost and high benefit. Based on the analysis of the WTP threshold line (diagonal dashed line) in the figure, it can be concluded that when the WTP threshold standard is 80,976 yuan (one time the *per capita* GDP), 84.20% of the scatter plot of ICUR values are located below the WTP threshold line. This indicates that 84.20% of the model simulation results have a higher cost-effectiveness value than those of non-screening (conventional healthcare).

## 5 Discussion

### 5.1 Analysis of depression status in pregnant and postpartum women in China

In recent years, multiple studies have shown that women have an increased risk of depression throughout the entire pregnancy and childbirth period, and the incidence of depression during pregnancy is not lower than PPD (5). The first part of this study was a multi-center, longitudinal follow-up cohort study based on the same group of pregnant and postpartum women. It aimed to understand the depression status of pregnant and postpartum women in China at different stages of the whole pregnancy and childbirth period and the natural outcomes at different time points.

The results showed that the respondents had depression risks in early pregnancy, mid pregnancy, late pregnancy, and postpartum, among which the detection rate of depression symptoms in early pregnancy was the highest, the rate in late pregnancy was lower, and the rate postpartum was higher. This finding shows that there are differences in the depression status of Chinese pregnant women at different time points during pregnancy and childbirth, with a higher incidence in the early stages of pregnancy. Given the heterogeneity of depression throughout pregnancy and the postpartum period, multiple evaluation periods are of great value in supporting maternal mental health. These findings also suggest that conducting depression screening in the early stages of pregnancy, identifying high-risk pregnant women, and intervening early can prevent the continuation or progression of depression in the later stages, improve women’s experience of pregnancy, and reduce the occurrence of adverse pregnancy outcomes.

### 5.2 Effectiveness of screening strategies for depression during pregnancy and childbirth

The depression status of pregnant and postpartum women who have undergone a screening intervention is not significantly different from that of pregnant and postpartum women who only receive routine health care. However, the positive detection rate of depression in early pregnancy is significantly reduced after screening, and the postpartum positive detection rate is reduced by 14.7%. This indicates that timely screening, identifying high-risk pregnant women, and providing necessary intervention and treatment at each critical period can effectively prevent the continuation of depressive symptoms, ultimately reducing the occurrence of depressive states. This is also the fundamental purpose of conducting prenatal depression screening.

### 5.3 Methodological considerations

As in most international studies, the screening strategy evaluated in this study included corresponding intervention measures, and there was no separate cost calculation and economic evaluation of the screening itself. We set up a Markov state based on the outcomes of depression at different time points during pregnancy and childbirth in order to demonstrate the different outcomes. This situation is more in line with the characteristics of the true outcomes of depression during pregnancy and childbirth. The cost-effectiveness analysis results indicated that from a societal perspective screening strategies have cost-effectiveness and a dominant position compared with non-screened (routine care). Sensitivity analysis also showed the robustness of the results of this study.

To the best of our knowledge, no published studies exist on health economics related to prenatal depression screening both in China and internationally. Studies on a similar topic mostly focus on the health economics evaluation of PPD screening and intervention, with less involvement in pregnancy or the entire pregnancy and childbirth period. The conclusion of this study was similar to that of Premji et al. (32) who used the perspective of the healthcare system and decision trees to evaluate the cost-effectiveness of PPD screening and non-screening (conventional healthcare). Premji et al. (32) conducted two analyses, current practice (51% of referrals) and scenario analysis (100% of referrals), and the results showed that screening is most valuable when participation and compliance are maximized, as all high-risk women screened participated in referrals. Wilkinson et al. (33) used decision trees to simulate the cost-effectiveness of collaboration between doctors and psychiatrists in screening and treating PPD. These researchers simulated a 2-year duration and found that screening is cost-effective compared with non-screening (conventional healthcare), similar to our conclusions. Paulden et al. (34) constructed a decision tree model for screening, non-screening, and treatment, considering the identification, treatment, and possible recurrence of PPD as a complete treatment path. They evaluated the cost-effectiveness of postpartum scale screening from 6 weeks to 1 year postpartum. However, this study only considered the cost increase of screening alone and did not consider screening and intervention as a whole; therefore, the results suggested that simple scale screening did not have economic value.

### 5.4 Public health implications and future suggestions

Depression during pregnancy and PPD are highly prevalent and have negative long-term effects on mothers, families, and society. Many developed countries have recommended developing strategies to regularly provide psychological screening services for mothers, while the field of maternal mental health in China is still in its developmental stage. The specific measures regarding screening tools, screening timing, frequency, and other factors still need verification. Many pregnant and postpartum women experience psychological problems during pregnancy and childbirth, but general understanding still needs to be improved as this issue has not received sufficient attention and women lack effective ways to seek help. Considering the scarcity of Chinese psychiatrists and the special nursing needs of the pregnant and postpartum population, it is currently the most cost-effective way to conduct prenatal depression screening and provide initial intervention support in maternal and child health centers and other obstetric and gynecological institutions. Current results indicate that screening for depression during pregnancy and childbirth is better than not screening; therefore, medical staff from midwifery institutions who have the most contact with pregnant women should consider conducting regular screening. Timely identification and treatment have been shown to reduce the incidence of depression during pregnancy and childbirth (35, 36), which in turn enables pregnant women and mothers to enjoy a higher quality of life, leading to healthier lifestyles, higher work efficiency, greater participation in family life, and reduced adverse consequences for children (37, 38).

Combining the results of on-site investigation and sensitivity analysis, we found that reducing screening costs is the most effective way to improve screening rates and the overall economic value of screening strategies. Specific suggestions for reducing screening costs include the following:

1. Government packaged procurement services will effectively reduce costs and improve screening effectiveness. Local government support can create a favorable environment for the implementation of screening strategies, while reducing screening costs and improving screening effectiveness.
2. An integrated screening strategy helps to reduce screening costs. Different regions in China have different development conditions, and this study only made a comparison between a screening strategy and non-screening (routine health care). Each region can develop its own screening program for depression in pregnancy and childbirth according to the actual situation and carry out validation and evaluation studies. At present, China’s combination of prenatal examination time and postnatal visit time is also a good program, so the screening execution method can be a combination of existing clinical work without adding additional clinical burden.
3. The electronic screening method helps to reduce the screening cost. As for the survey method, the current screening methods of the survey institutions include completing the scale screening on the computer in the psychological department, a paper screening questionnaire, mobile phone app, official WeChat, and other methods. Electronic information channels may be a more economical and efficient way to proceed, and in the future, specific comparative studies on screening methods, screening frequency, and screening time points can be continued to determine the optimal screening strategy.

## 6 Limitations

A potential limitation of this study is that the model involved multiple sources of data, including queue data from previous studies and data from the latest survey. Additionally, literature evidence was used to supplement data that could not be evaluated in a short period of time. To mitigate these potential limitations, the costs were tested in sensitivity analyses, which indicated that the variability of the screening costs did not significantly affect ICER.

This study only considered the cost of treating depression during pregnancy and childbirth from the perspective of the healthcare system, which to some extent underestimates the total cost of depression during pregnancy and childbirth. We emphasize that the cost-effectiveness analysis of this study is conservative as it only considers the impact of perinatal depression during the first year (pregnancy and postpartum) and does not include the broad negative effects of perinatal depression on women, children, and their families in the short and long term. Future research should also expand the model. Ideally, the economic costs associated with depression during pregnancy and childbirth would be estimated from a broad societal perspective. This would increase the direct non-medical costs (such as transportation and childcare costs) and indirect costs (such as productivity losses) associated with the disease, including costs incurred by fathers (39, 40) and other family members. Incorporating economic measures into longitudinal studies for long-term follow-up could also evaluate sequelae in mid to later childhood.

## 7 Conclusions and recommendations

This cost-effectiveness analysis of the currently recommended prenatal depression screening strategies in China suggests that from a societal perspective, prenatal depression screening strategies may have cost-effectiveness. Our results show that the implementation of screening strategies can provide a good cost performance ratio for depression during pregnancy and childbirth and improve QALY. Based on these results, medical decision-makers should be encouraged to implement and manage recommended depression screening strategies for pregnant and postpartum women, and by doing so, to maximize the effective allocation of resources.

## Data availability statement

The data analyzed in this study is subject to the following licenses/restrictions: This study is only based on analysis results and does not reflect any case data. Requests to access these datasets should be directed to YY, yangyehuan@chinawch.org.cn.

## Ethics statement

The studies involving humans were approved by Ethical Review Committee of National Center for Women and Children's Health of Chinese Center for Disease Control and Prevention. The studies were conducted in accordance with the local legislation and institutional requirements. Written informed consent for participation was not required from the participants or the participants' legal guardians/next of kin in accordance with the national legislation and institutional requirements.

## Author contributions

YY: Data curation, Investigation, Methodology, Writing – original draft, Writing – review & editing. RZ: Conceptualization, Funding acquisition, Investigation, Methodology, Project administration, Resources, Supervision, Writing – review & editing. LY: Data curation, Investigation, Methodology, Project administration, Supervision, Validation, Writing – review & editing. XH: Data curation, Investigation, Project administration, Writing – review & editing. TZ: Methodology, Resources, Supervision, Writing – review & editing.
